# MVnet: automated time-resolved tracking of the mitral valve plane in CMR long-axis cine images with residual neural networks: a multi-center, multi-vendor study

**DOI:** 10.1186/s12968-021-00824-2

**Published:** 2021-12-02

**Authors:** Ricardo A. Gonzales, Felicia Seemann, Jérôme Lamy, Hamid Mojibian, Dan Atar, David Erlinge, Katarina Steding-Ehrenborg, Håkan Arheden, Chenxi Hu, John A. Onofrey, Dana C. Peters, Einar Heiberg

**Affiliations:** 1 Clinical Physiology, Department of Clinical Sciences, Lund University, Skåne University Hospital, Lund, Sweden; 2 Department of Radiology and Biomedical Imaging, Yale School of Medicine, Yale University, New Haven, Connecticut United States of America; 3 Department of Electrical Engineering, Universidad de Ingeniería y Tecnología, Lima, Peru; 4 Department of Biomedical Engineering, Lund University, Lund, Sweden; 5 Department of Cardiology B, Oslo University Hospital Ullevål and Faculty of Medicine, University of Oslo, Oslo, Norway; 6 Department of Cardiology, Clinical Sciences, Lund University, Skåne University Hospital, Lund, Sweden; 7 School of Biomedical Engineering, Shanghai Jiao Tong University, Shanghai, China; 8 Department of Urology, Yale School of Medicine, Yale University, New Haven, Connecticut United States of America; 9 Department of Biomedical Engineering, Yale University, New Haven, Connecticut United States of America; 10 Wallenberg Center for Molecular Medicine, Lund University, Lund, Sweden

**Keywords:** Left ventricular dysfunction, Annotation, Residual neural networks

## Abstract

**Background:**

Mitral annular plane systolic excursion (MAPSE) and left ventricular (LV) early diastolic velocity (e’) are key metrics of systolic and diastolic function, but not often measured by cardiovascular magnetic resonance (CMR). Its derivation is possible with manual, precise annotation of the mitral valve (MV) insertion points along the cardiac cycle in both two and four-chamber long-axis cines, but this process is highly time-consuming, laborious, and prone to errors. A fully automated, consistent, fast, and accurate method for MV plane tracking is lacking. In this study, we propose MVnet, a deep learning approach for MV point localization and tracking capable of deriving such clinical metrics comparable to human expert-level performance, and validated it in a multi-vendor, multi-center clinical population.

**Methods:**

The proposed pipeline first performs a coarse MV point annotation in a given cine accurately enough to apply an automated linear transformation task, which standardizes the size, cropping, resolution, and heart orientation, and second, tracks the MV points with high accuracy. The model was trained and evaluated on 38,854 cine images from 703 patients with diverse cardiovascular conditions, scanned on equipment from 3 main vendors, 16 centers, and 7 countries, and manually annotated by 10 observers. Agreement was assessed by the intra-class correlation coefficient (ICC) for both clinical metrics and by the distance error in the MV plane displacement. For inter-observer variability analysis, an additional pair of observers performed manual annotations in a randomly chosen set of 50 patients.

**Results:**

MVnet achieved a fast segmentation (<1 s/cine) with excellent ICCs of 0.94 (MAPSE) and 0.93 (LV e’) and a MV plane tracking error of −0.10 ± 0.97 mm. In a similar manner, the inter-observer variability analysis yielded ICCs of 0.95 and 0.89 and a tracking error of −0.15 ± 1.18 mm, respectively.

**Conclusion:**

A dual-stage deep learning approach for automated annotation of MV points for systolic and diastolic evaluation in CMR long-axis cine images was developed. The method is able to carefully track these points with high accuracy and in a timely manner. This will improve the feasibility of CMR methods which rely on valve tracking and increase their utility in a clinical setting.

**Supplementary Information:**

The online version contains supplementary material available at 10.1186/s12968-021-00824-2.

## Background

The mitral valve (MV) is a fibrous region that separates the left ventricle (LV) and the left atrium with two leaflets. In the normal heart, the MV remains closed during systole and the MV plane rapidly descends with contraction of the ventricles; in early diastole the MV opens, and the MV plane quickly springs back to an equilibrium plane where it pauses during diastasis, and then ascends further during the left atrial kick in late diastole. The analysis of MV plane motion provides structural and functional systolic and diastolic information [1]. Its measurement yields peak displacement of the plane during systole, known as mitral annular plane systolic excursion (MAPSE), and the LV early diastolic velocity, known as LV e’, which is itself a key metric of diastolic function in echocardiography [2].

Cardiovascular magnetic resonance (CMR) is a reproducible imaging modality and is considered the reference standard for cardiac volume assessment. Its accuracy and reliability hold promise for serial examinations of MAPSE and LV e’, as already reported in our previous work [3]. These metrics were obtained by tracking the MV insertion points in every frame in long-axis cine images. MV plane tracking has also been used to enable slice-following for assessment of valvular flow with a phase-contrast sequence, either retrospectively [4] or prospectively [5], where it allows an evaluation of mitral regurgitation, which would not be possible without valve tracking. The MV plane location can assist any automated segmentation of the LV or left atrium, as it demarcates these chambers [6], and its tracking can provide an estimate of global longitudinal strain [7]. Finally, MV plane dynamics could be useful in providing information on the timing of the cardiac rest-periods, which is important in CMR [8–10].

Even with significant improvements in semi-automated tracking methods of the MV points in cine CMR [11–16], and validation of clinical metrics against echocardiography [3, 16], MV plane tracking still requires a manual initialization and refinement. A fully automated, fast and accurate method for tracking the MV points using standard clinical CMR images is lacking. The rapid evolving field of deep learning has great potential to provide such method. In CMR deep learning applications [17], and deep learning in general, it is strongly suggested to have a large cohort of training data coming from different centers, vendors, pathologies, and manually labeled by different experts. Such diversity in the data leads to a consistent, robust method to perform a determined task.

In this study, we develop MVnet, a dual-stage deep learning approach for MV tracking using residual neural networks, trained and tested in a multi-center, multi-vendor population of 703 patients, with a wide range of pathologies, and manually labeled by 10 experts. Additionally, we show the importance of using a rich training dataset by structurally analyzing two main scenarios with direct impact on the learning and application: a single-center, single-vendor, single-expert dataset compared to a multi-center, multi-vendor, multi-expert dataset. We also evaluate the derived clinical parameters (MAPSE and LV e’) in contrast with their counterpart by experts. Finally, with this dual-stage pipeline, we present technical novelty by applying an automated linear transformation to the images after the first stage, which substantially reduces variance and boosts performance in accuracy.

## Methods

### Imaging data

A multi-center, multi-vendor population of 703 subjects (226 females, 51±19 years old) for diverse clinical indications were retrospectively enrolled, as part of IRB approved chart-review studies. The reported pathologies (Table 1) included subjects with myocardial infarction (n = 169), chronic heart failure (n = 130), arrhythmia - mostly atrial fibrillation (n = 63), heart failure with reduced LV ejection fraction (n = 54), endurance athletes (n = 39), pulmonary arterial hypertension (n = 25), atrial septal defect (n = 19), hypertrophic cardiomyopathy (n = 14), other cardiac diseases (n = 13), sarcoid (n = 6), myocarditis (n = 6), and healthy volunteers of all ages (n = 165). All subjects were scanned on a 1.5T (n = 661) or a 3T (n = 42) conventional clinical CMR scanners from Philips Healthcare (Best, the Netherlands; n = 419), Siemens Healthineers (Erlangen, Germany; n = 250), and General Electric Healthcare (Chicago, Illinois, USA; n = 34). Inclusions were performed in 16 different centers and 7 countries, and included standard two-chamber and four-chamber long-axis cine exams, according to clinical practice. Typical image parameters were breath-hold with repetition time of 2.8 to 3.0 ms, echo time of 1.4 to 1.5 ms, and flip angle of 50 to 60°. Imaging data of both chamber views had a spatial resolution ranging from 1.3 $\times$ 1.3 mm^2^ to 1.7 $\times$ 1.7 mm^2^ (after zero-filling), slice thickness of 8 mm and typically 30 (25 to 50) temporal frames per cardiac cycle were reconstructed. The final dataset comprised a total of 38,854 images (703 sets of time-resolved images) analyzed in previous studies at Yale University (Yale dataset) [3, 6, 18, 19] and at Lund University (Lund dataset) [20–33].

**Table 1:** Multi-vendor, multi-center database (n = 703) description by pathology, gender and age

Dataset	Vendor	Center	Pathological group	n	Female	Age
Yale (n = 150)	Siemens (n = 150)	Yale New Haven Hospital (USA)	Arrythmia	63	18	54 ± 15
			Pulmonary arterial hypertension^†^	25	18	62 ± 14
			Hypertrophic cardiomyopathy	14	4	51 ± 15
			Sarcoid	6	5	52 ± 15
			Myocarditis	6	3	41 ± 15
			Myocardial infarction	4	1	43 ± 13
			Other cardiac diseases	13	7	51 ± 13
			Healthy adult volunteers	19	5	51 ± 14
Lund (n = 553)	Philips (n = 419)	CHILL-MI and MITOCARE* (4 centers, 3 countries)	ST-elevation myocardial infarction	57	14	53 ± 19
		Skåne University Hospital (Sweden)	Chronic heart failure	130	28	60 ± 13
			Heart failure with reduced ejection fraction	54	11	68 ± 9
			Atrial septal defect	19	13	50 ± 18
			Endurance athletes	39	11	39 ± 18
			Healthy adolescent volunteers	39	21	13 ± 2
			Healthy young adult volunteers^†^	57	25	26 ± 5
			Healthy senior adult volunteers	24	10	53 ± 8
	Siemens (n = 100)	CHILL-MI and MITOCARE* (13 centers, 4 countries)	ST-elevation myocardial infarction	74	19	50 ± 2
		Skåne University Hospital (Sweden)	Healthy young adult volunteers	7	2	27 ± 2
			Healthy senior adult volunteers	19	6	56 ± 5
	General Electric (n = 34)	MITOCARE* (2 centers, 2 countries)	ST-elevation myocardial infarction	34	5	62 ± 9

The mean ± standard deviation are reported for age.

Subjects were scanned at 1.5T magnetic resonance scanners (n = 661).

^†^ 25 patients with pulmonary arterial hypertension and 17 healthy young adult volunteers were scanned at 3T magnetic resonance scanners.

* CHILL-MI study comprised 85 patients, MITOCARE study comprised 80 patients. Note that some subjects were excluded compared to the original trials as long-axis images were missing. Duplicates with data from Skåne University Hospital were removed as this was one of the centers

### Manual annotation

**Fig. 1:**
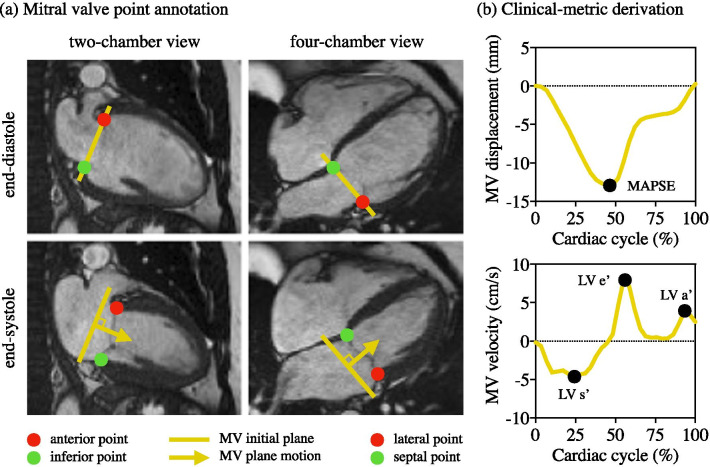
**a** MV point annotation illustration at end-diastole and end-systole in 2-chamber view, with anterior and inferior points, and 4-chamber view, with lateral and septal points, and **b** the clinical-metric derivation as an output of the time-resolved annotation. The MV displacement was measured as the average of the perpendicular distances from the MV initial plane, defined at end-diastole in each view, to every MV point at every temporal frame, and the MV velocity was measured as its time-derivative. MAPSE was calculated as the maximum displacement, and LV s’, e’ and a’ as the first, second and third global velocity peak. *MV* mitral valve, *MAPSE* mitral annular plane systolic excursion, *LV* left ventricle

Using an analysis tool, freely available in the software Segment [34], 10 trained human observers with different backgrounds and levels of CMR experience, ranging from 4 to 20 years, performed these annotations in all temporal frames of 458 subjects and only at end-diastolic and end-systolic frames of 245 subjects (belonging to the Lund dataset). The experts placed the MV insertion points in two-chamber view data, as anterior and inferior points, and in four-chamber view data, as lateral and septal points, at each phase in the cardiac cycle, resulting in two points for each view in each image (Fig. 1a).

With no society recommendations on how to annotate and track the MV points, the Yale and Lund datasets were annotated according to two different principles. A single observer at Yale University (Yale observer) placed the MV points in the intersection between the MV and the LV myocardium, whereas the 9 observers at Lund University (Lund observers) placed the MV points at the most basal part of the compact LV myocardium. Both ways yielded similar MV motions but with a different reference point. However, due to the inherent observer bias and the high number of Lund observers, these annotations presented more variability. Such differences in manual annotation are very common in deep learning applications.

### Dual-stage residual neural network

**Fig. 2:**
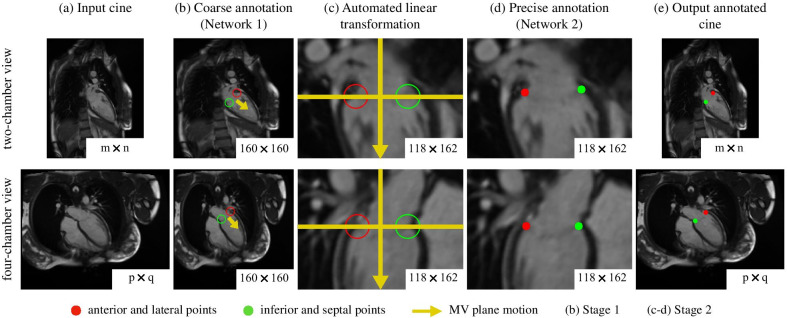
MVnet pipeline. **a** The input cine images with an inherent clinical variability (size $m \times n$, resolution, orientation and cropping) were fed to the proposed dual-stage residual neural network. **b** The first trained ResNet-50 produced coarse annotations, marked in circumferences representing acceptable accuracy, in every cine image in a fixed image size of 160 $\times$ 160, which in turn, served to apply a **c** linear transformation to a standard spatial resolution of 0.75 mm, orientation and cropping around the mitral valve center for a size of 118 $\times$ 162. **d** The second trained ResNet-50 used the transformed images to predict precise annotations, marked in circles representing higher accuracy, which were adjusted again to the original input image. These last two tasks could be done iteratively as indicated. **e** The output time-resolved coordinates were used to derive the mitral valve displacement and velocity curves

**Fig. 3:**
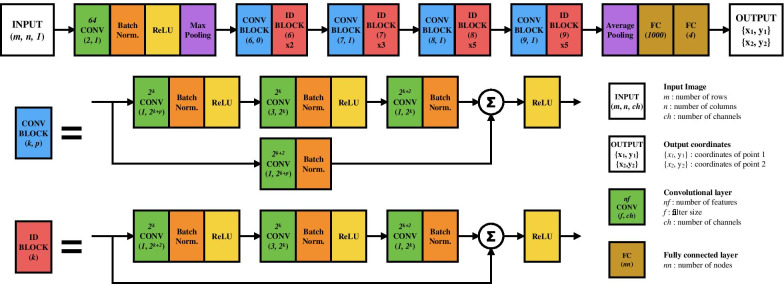
Adapted convolutional neural network architecture with a residual learning framework, ResNet50 [35], used in this study for automated mitral valve point tracking. With a given input image with size $m \times n$, the network outputs two mitral valve points $\{x_1, y_1, x_2, y_2\}$ in Cartesian coordinates

Based on our recent work to track the tricuspid valve [19] as proof of principle, we adapted and expanded our dual-stage residual neural network to track the MV. The proposed framework (Fig. 2) involved two stages for each chamber view. The first stage uses a trained network to track the MV points with sufficient accuracy to define the MV plane, and the second stage uses these points to perform a linear transformation on the original images to standardize the images regarding location and orientation of the valve plane. The second network then predicts the MV points in the automatically preprocessed image with higher accuracy. Each stage uses an artificial neural network with a residual framework of 50 layers, ResNet-50 [35], adapted to predict a series of four numbers (representing two pairs of coordinates $\{x,y\}$) on an individual grayscale cine image (Fig. 3).

**Fig. 4:**
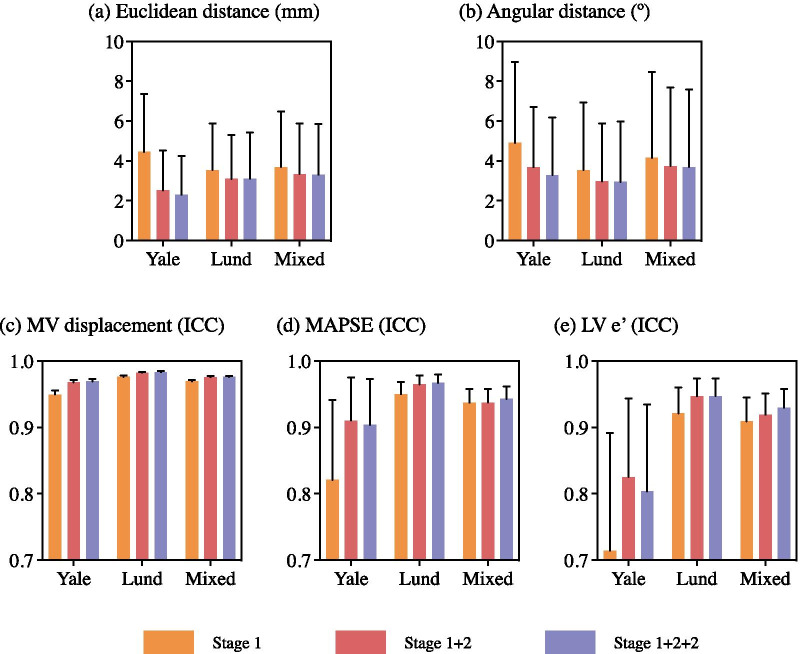
Accuracy of each model (MVnet) trained and evaluated on its own dataset, by the mean **a** Euclidean and **b** angular distance error, and the agreement with ICC in **c** MAPSE, **d** LV e’ and **e** MV displacement, stratified by the output of the first stage (stage 1), second stage (stage 1+2), and an iteration of the second stage (stage 1+2+2). For (**a**, **b**), each bar represents the mean, and error bar the standard deviation of each accuracy metric. For (**c**, **d**, **e**), each bar represents the ICC, and error bar the confidence interval (95%) of each accuracy metric. The output of the iteration (stage 1+2+2) achieved the best accuracy and was chosen for the proposed workflow. *ICC* intra-class correlation coefficient, *MAPSE* mitral annular plane systolic excursion, *LV* left ventricle, *MV* mitral valve

**Fig. 5:**
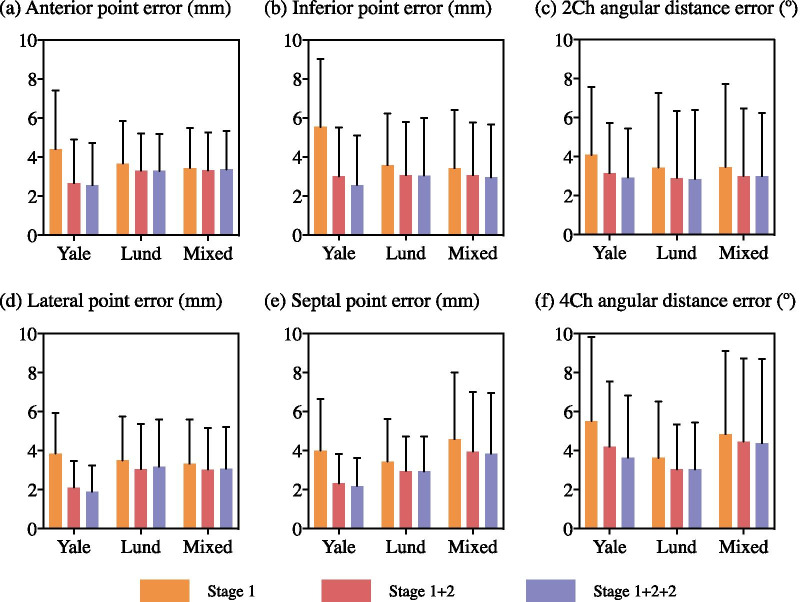
Accuracy of each model (MVnet) trained and evaluated on its own dataset, by the mean Euclidean distance error (first two columns) and angular distance error (third column), stratified by the output of the first stage (stage 1), second stage (stage 1+2), and an iteration of the second stage (stage 1+2+2). Accuracy assessed for 2Ch in its **a** anterior and **b** inferior point distance error, and **c** angular distance error; and for 4Ch in its **d** left ventricular lateral and **e** septal point distance error, and **f** angular distance error. Each bar represents the mean, and error bar the standard deviation of each accuracy metric. *2Ch* two-chamber view, *4Ch* four-chamber view

**Fig. 6:**
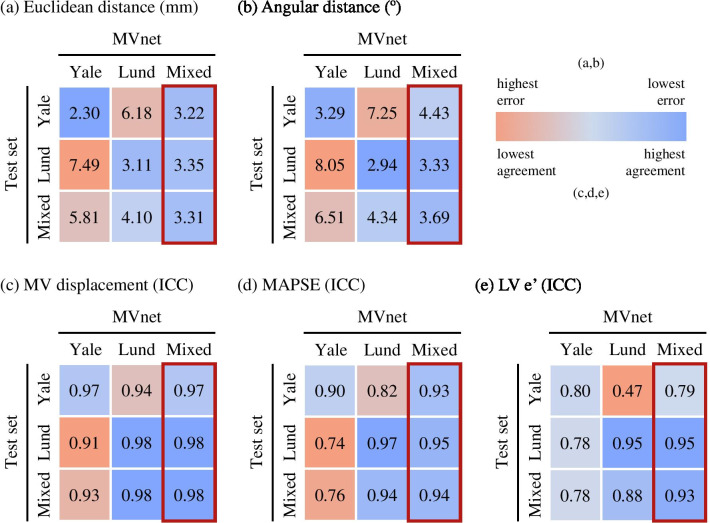
Accuracy heatmap of each model (MVnet) trained on each training set and evaluated on each test set by the mean **a** Euclidean and **b** angular distance error, and the agreement with ICC in **c** MV displacement, **d** MAPSE, and **e** LV e’. The output of ${{\text{MVnet}}_{\text{Mixed}}}$ consistently achieved the best accuracy and was chosen for the proposed model. *ICC* intra-class correlation coefficient, *MV* mitral valve, *MAPSE* mitral annular plane systolic excursion, *LV* left ventricle

**Fig. 7:**
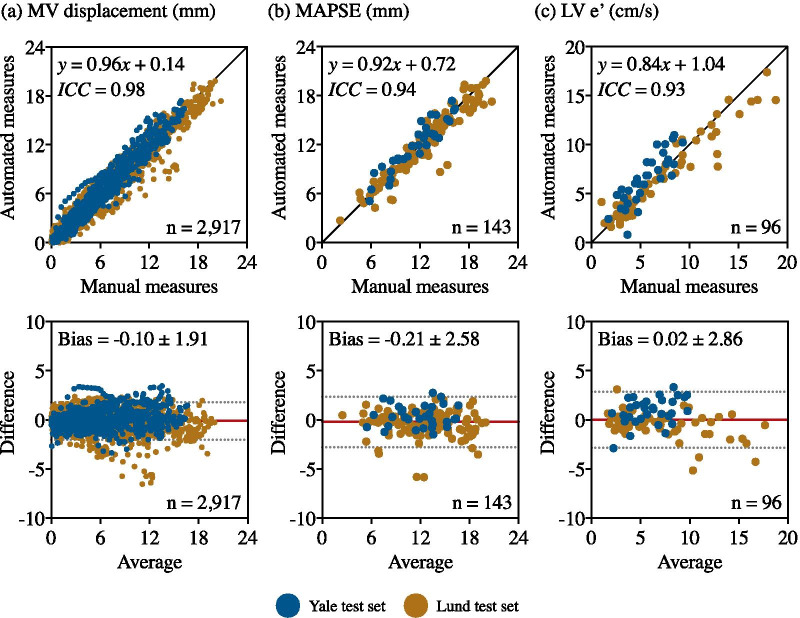
Clinical-metric agreement of **a** MV displacement, **b** MAPSE and **c** LV e’ between an expert manual annotation and the automated method (${{\text{MVnet}}_{\text{Mixed}}}$). The first row of each analysis shows the regression plots whereas the second shows the Bland-Altman plots. In each scatter plot the black line denotes the identity line, whereas in each Bland-Altman plot, the red line denotes the mean difference (bias) and the two light dotted lines denote ± 1.96 standard deviations from the mean. *MV* mitral valve, *MAPSE* mitral annular plane systolic excursion, *LV* left ventricle

**Fig. 8:**
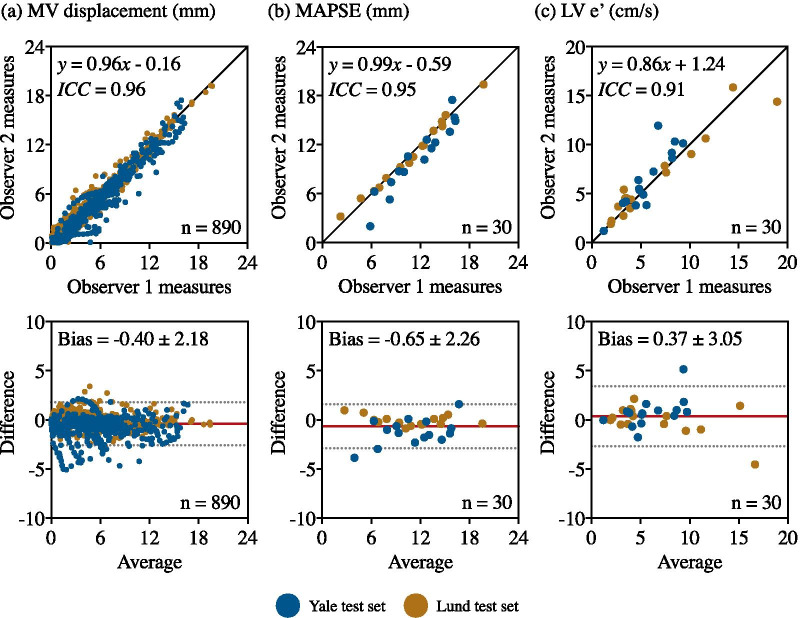
Clinical-metric agreement of **a** MV displacement, **b** MAPSE and **c** LV e’ between an expert manual annotation (observer 1) and a pair of second observers (observer 2). One observer from the Yale dataset and another from the Lund dataset annotated 25 subjects from each test set. The first row of each analysis shows the regression plots whereas the second shows the Bland-Altman plots. In each scatter plot the black line denotes the identity line, whereas in each Bland-Altman plot, the red line denotes the mean difference (bias) and the two light dotted lines denote ± 1.96 standard deviations from the mean. *MV* mitral valve, *MAPSE* mitral annular plane systolic excursion, *LV* left ventricle

#### Stage 1

The first stage only involves the use of one network to perform a coarse annotation in all temporal frames. The network was trained on manually annotated images resized to 160 $\times$ 160 with cubic interpolation, leading to anisotropic pixel dimensions. This initial coarse annotation serves to localize the MV plane and follow its motion.

#### Stage 2

The second stage uses the output coordinates of the previous stage to apply a linear transformation task, i.e., standardize the cine, by (i) interpolating each image to a spatial isotropic resolution of 0.75 mm, (ii) rotating each image such that the MV is oriented horizontally with the apex pointing down and with the anterior and lateral points placed on the left and the inferior and septal points placed on the right, for two-chamber and four-chamber views, respectively, and (iii) cropping each image around the MV center for a size of 118 $\times$ 162.

#### Pipeline

As an overview, a given input cine of a chamber view with different parameters (Fig. 2a) is fed into the first stage with a fixed size for the network to read (Fig. 2b). The first trained network (Fig. 3) outputs the predicted points with acceptable accuracy. The coarse annotation on the first temporal frame is used as a reference to determine the orientation and centering tasks, i.e., to horizontally orient the MV in the center, whereas the coarse annotation on the remaining temporal frames determine the motion direction, i.e., to ensure the apex points down. The automated linear transformation task from the second stage standardizes the spatial resolution, the heart orientation and positioning and the type of cropping (Fig. 2c). The second network (Fig. 3), trained on linearly transformed images, is then used to track the MV points with high accuracy (Fig. 2d). These new predicted points are readjusted, with an inverse standardization transformation, to match the original input cine images (Fig. 2e). This stage can be performed in an iterative manner as indicated, i.e, the points predicted by the second stage can initialize again the second stage (linear transformation task and second network) to yield a more accurate annotation.

#### Network training

Both networks were trained with the same process but independently for each stage and chamber view. Transfer learning for weights initialization was applied to reduce convergence time and aid the learning process, using a ResNet-50 pretrained on more than one million images from the ImageNet database [36] for a classification task into 1000 different categories of objects and animals photographs. Standard data preparation involved pixel distribution normalization by the median and interquartile range to ensure generalizability [37]. Training data was augmented 10 times by scaling ±10%, rotating ±10$^{\circ }$ and translating ±3 pixels to add more inherent variability in the first stage, and to compensate for any error introduced from the first stage to the second stage, i.e., a slight misalignment of the MV plane center from the ground truth. The mean square error loss function was optimized by the Adam method [38] with a learning rate of $1\times 10^{-4}$, for 20 epochs and mini-batch size of 8. The pipelines were developed on MATLAB R2019b (Mathworks, Natick, MA) with a NVIDIA Titan RTX GPU.

### Clinical metric derivation

The MV plane displacement was calculated for each chamber view as the average perpendicular distance of the MV points to the initial plane set in end-diastole (Fig. 1b). The resultant MV plane displacement was measured as the average from both chamber views. MAPSE was derived from the maximal MV displacement and LV e’ was the second global peak of the time-derivative of the displacement curve.

### Evaluation

#### Data variability analysis

Both Yale and Lund datasets were trained and tested separately and collectively to assess the influence of employing a single-center, single-vendor, single-expert dataset (Yale) and a multi-center, multi-vendor, multi-expert dataset (Lund). This analysis was performed in three different pipelines: ${{\text{MVnet}}_{\text{Yale}}}$, ${{\text{MVnet}}_{\text{Lund}}}$ and ${{\text{MVnet}}_{\text{Mixed}}}$, for the Yale dataset, Lund dataset, and both mixed, respectively. Each MVnet comprised of 4 networks in total, i.e., 2 networks for the first and second stage of the two-chamber view and other 2 networks for both stages of the four-chamber view. For ${{\text{MVnet}}_{\text{Yale}}}$, the training and testing sets were partitioned into 6948 images (118 subjects) and 1886 images (32 subjects), respectively. For ${{\text{MVnet}}_{\text{Lund}}}$, the training and testing sets were partitioned into 26,072 images (118 subjects) and 3948 images (111 subjects), respectively. For ${{\text{MVnet}}_{\text{Mixed}}}$, the same distribution set for each dataset was used and mixed. Such distributions (Table 2) were randomly performed with a constraint to have an homogeneous representation of the reported cardiovascular diseases.

**Table 2:** Distribution of training and test sets of annotated cine images from each dataset

Dataset	Division	Training set	Test set	Total
Yale	Subjects	118	32	150
	2Ch cine images	3498	943	4441
	4Ch cine images	3450	943	4393
	Total cine images	6948	1886	8834
Lund	Subjects	442	111	553
	2Ch cine images	13,018	1974	14,992
	4Ch cine images	13,054	1974	15,028
	Total cine images	26,072	3948	30,020
Mixed	Subjects	560	143	703
	2Ch cine images	16,516	2917	19,433
	4Ch cine images	16,504	2917	19,421
	Total cine images	33,020	5834	38,854

*2Ch* two-chamber view, *4Ch* four-chamber view

#### Spatial annotation accuracy

The test set of each MVnet was evaluated against manual annotation. The spatial annotation error was measured in each chamber view with: (i) the Euclidean distance, which measured in millimeters the distance error between the ground-truth and predicted annotations, (ii) the angular distance, which measured in degrees the inner intersection angle of the ground-truth and automated planes defined by both MV points, and (iii) the MV displacement, which measured in millimeters the difference in the MV plane displacement in every temporal frame between the ground-truth and automated displacement curves. All metrics were calculated comparing the ground-truth with the predicted annotations on the original input images.

#### Clinical-metric accuracy

Clinical metric (MAPSE and LV e’) comparisons were performed using linear regression analysis, Bland-Altman plots, and the intra-class correlation coefficient (ICC) between the automated and manual measures. As 47 subjects (out of 111) from the Lund test set were only annotated at end-diastole and end-systole, minimum amount of temporal frames required for MAPSE, LV e’ comparisons were not performed in that subset. The threshold for statistical significance was considered to be p<0.05 for this study.

#### Dual-stage influence

Both spatial annotation and clinical-metric accuracy evaluations were performed for the results of the first stage (stage 1), both stages (stage 1 + 2) and an additional iteration (stage 1 + 2 + 2), to show the influence of the additional stage (Fig. 2c, d).

#### Inter-observer variability analysis

For inter-observer variability analysis, an additional pair of observers performed manual annotations in a randomly chosen of 50 subjects. Specifically, a second observer of Yale dataset (Yale observer 2) and Lund dataset (Lund observer 2) manually annotated a subset of 25 subjects each on its corresponding dataset and the same evaluation was assessed.

## Results

### Implementation

MVnet was implemented in the medical image analysis software Segment v3.1 R8109 [34] (http://segment.heiberg.se), which is freely available for research purposes, and uploaded to https://github.com/ra-gonzales/MVnet. Total training time, including each stage and chamber view, took 108, 260 and 420 hours for ${{\text{MVnet}}_{\text{Yale}}}$, ${{\text{MVnet}}_{\text{Lund}}}$ and ${{\text{MVnet}}_{\text{Mixed}}}$, respectively. For each ${\text{MVnet}}$, on the GPU, testing time took 5.2 seconds per patient, whereas on a CPU, it took 10.8 seconds per patient, including data I/O time, compared to an average manual annotation time from 8 to 20 minutes. Batch processing reduces the average time to under 1 second on the GPU.

### Dual-stage influence

The annotation accuracy of each $\text {MVnet}$ after the first stage (stage 1), the second stage (stage 1+2) and an iteration of the second stage (stage 1+2+2) is reported in Fig. 4, in terms of spatial annotation and clinical-metric accuracy, between every ground-truth and predicted measures of an $\text {MVnet}$ with its corresponding test set.

In terms of Euclidean and angular distance agreement (Fig. 4a, b), the mean percentage error of both metrics from the first to the second stage decreased 34%, 14%, and 10% for ${{\text{MVnet}}_{\text {Yale}}}$, ${{\text{MVnet}}_{\text{Lund}}}$ and ${{\text{MVnet}}_{\text {Mixed}}}$, respectively. In a similar manner, the overall error from the first stage to the second iteration of the second stage decreased 41%, 15%, and 11%, respectively. Accuracy and agreement were consistently improved after each stage. No substantial differences were found in a specific MV point and chamber view (Fig. 5), meaning they all achieved a similar accuracy. However, the angular distance error on the four-chamber view was larger with a higher discordance in the septal point placement from the difference in the two annotation principles.

Regarding MV displacement, MAPSE and LV e’ agreement (Fig. 4c–e), with the addition of the second stage, the mean percentage error decreased 42%, 29%, and 11% for ${{\text{MVnet}}_{\text{Yale}}}$, ${{\text{MVnet}}_{\text{Lund}}}$ and ${{\text{MVnet}}_{\text{Mixed}}}$, respectively, whereas the iteration of the second stage reduced the initial error 39%, 32%, and 18%. This iteration improved the agreement with clinical metrics, except for ${{\text{MVnet}}_{\text{Yale}}}$ which showed a small reduction in agreement. Although the improvement of the iteration was moderate in the spatial annotation accuracy for ${{\text{MVnet}}_{\text{Lund}}}$ and ${{\text{MVnet}}_{\text{Mixed}}}$, the clinical-metric accuracy was further improved.

### Dataset variability influence

With the output of each $\text {MVnet}$ considered to be the predictions after an iteration of the second stage (stage 1+2+2), the accuracy of every model evaluated on every test set (the Yale, Lund and mixed test sets) is reported as a heatmap for each metric in Fig. 6, where the best performance among the models is highlighted in blue and the worst in red. Although both ${{\text{MVnet}}_{\text{Yale}}}$ and ${{\text{MVnet}}_{\text{Lund}}}$ performed well on their corresponding datasets, with generally superior performance for Lund, the accuracy was noticeably reduced when the Yale (or Lund) network was applied to Lund (or Yale) test dataset, with Lund (or Yale) annotations. Then there was a 2 to 3 times-fold error increase in Euclidean and angular distances, mainly as a result of difference in annotation pattern. The MV displacement metric, however, achieved a better agreement in this scenario, but still with lower performance.

While ${{\text{MVnet}}_{\text {Yale}}}$ achieved the lowest Euclidean distance error (although trained on less data), its error on the Lund test set was 3.3 times higher. Vice versa, ${{\text{MVnet}}_{\text{Lund}}}$ on the Yale test set was also notably higher (2.4 times). Similarly, the clinical-metric agreement of a model tested in a different test set was markedly decreased. In the case of LV e’, ${{\text{MVnet}}_{\text{Lund}}}$ failed on predicting the same clinical values as the manual measures with an ICC of 0.47. In contrast, ${{\text{MVnet}}_{\text{Mixed}}}$ performed with the same consistency, and overall it achieved a better, more robust agreement with both groups of human experts.

### Clinical-metric accuracy

Choosing ${{\text{MVnet}}_{\text{Mixed}}}$ as the proposed pipeline, the accuracy of clinical metrics for MAPSE and LV e’ of the automated method compared against the manual metrics, evaluated in the mixed test set, are shown in Table 3. The model estimated both metrics with excellent agreement with a mean error of −0.2±1.3 mm (ICC = 0.94) and 0.0±1.5 cm/s (ICC = 0.93), for MAPSE and LV e’, respectively. The regression and Bland-Altman plots for the MV parameters between the automated and manual measurements are presented in Fig. 7, where an excellent correlation and good agreement were observed for each of the three parameters, including the MV displacement. All reported correlation values are significant (p<0.0001).

### Inter-observer variability analysis

The inter-observer clinical-metric agreement is shown in Table 4. The results were on par with the automated predictions, with a mean error of −0.3±1.2 mm (ICC = 0.95) and 0.3±1.7 cm/s (ICC = 0.89), for MAPSE and LV e’, respectively. In a similar manner, the regression and Bland-Altman plots for the clinical parameters between the second and first group of observers are presented in Fig. 8. All reported correlation values are also significant (p<0.0001).

### Spatial annotation accuracy comparison

The spatial annotation agreement of the automated annotations by ${{\text{MVnet}}_{\text{Mixed}}}$ against manual annotations as well as the inter-observer spatial variability are presented in Table 5. Interestingly, while the Euclidean and angular distance errors seem to lower the pipeline performance, the automated reproducibility of clinical metrics is very high resulting in a very low MV displacement error. This shows that tracking the motion is more reproducible, and more relevant, than tracking the spatial location of each individual point. Therefore, the individual distance errors are not necessarily a good metric for evaluating the performance of a valve plane movement and may be misleading when the accuracy of the annotation model is very high. This discrepancy was also present in the studied inter-observer variability analysis as the Yale observer 2 yielded a Euclidean distance error of 2.7 ± 2.6 mm against ground truth, whereas the error of the Lund observer 2 was 3.9 ± 3.0 mm. Although this difference may flag a potential pitfall in the manual annotation, the clinical-metric agreement showed the opposite as the latter achieved an average ICC = 0.97, whereas the former an average ICC = 0.82, indicating that annotation consistency along temporal frames prevails above a specific annotation pattern.

**Table 3:** Automated clinical metric accuracy of mitral valve derived parameters

Clinical agreement	n subjects	Manual measures	Automated measures	Error measures	ICC (CI 95%)
MAPSE (mm)	143	12.3 ± 4.0	12.1 ± 3.9	−0.2 ± 1.3	0.94 (0.92–0.96)
LV e’ (cm/s)	96	6.6 ± 4.1	6.6 ± 3.7	0.0 ± 1.5	0.93 (0.90–0.95)

The mean ± standard deviation are reported for manual and automated measures and their error, evaluated on the mixed test set with the proposed model (${{\text{MVnet}}_{\text{Mixed}}}$). *MAPSE* mitral annular plane systolic excursion, *LV *left ventricle, *ICC* intra-class correlation coefficient, *CI* confidence interval

**Table 4:** Manual inter-observer clinical metric accuracy of mitral valve derived parameters

Clinical agreement	n subjects	Manual measures	Observer 2 measures	Error measures	ICC (CI 95%)
MAPSE (mm)	50	11.1 ± 3.8	10.8 ± 3.9	−0.3 ± 1.2	0.95 (0.91–0.97)
LV e’ (cm/s)	50	6.1 ± 3.6	6.4 ± 3.5	0.3 ± 1.7	0.89 (0.81–0.93)

The mean ± standard deviation are reported for manual and automated measures and their error, evaluated with ${{\text{MVnet}}_{\text{Mixed}}}$ on the mixed test set. *MAPSE* mitral annular plane systolic excursion, *LV* left ventricle, *ICC* intra-class correlation coefficient, *CI* confidence interval

**Table 5:** Automated and manual inter-observer spatial annotation agreement

Spatial agreement	n images	Euclidean distance error (mm)	Angular distance error (º)	MV displacement error (mm)
${{\text{MVnet}}_{\text{Mixed}}}$	5834	3.31 ± 2.55	3.69 ± 3.89	**−0.10 ± 0.97**
Inter-observer	2960	3.29 ± 2.90	4.05 ± 4.22	**−0.15 ± 1.18**

The mean error ± standard deviation are reported for Euclidean and angular distances, and mitral valve displacement error between ground-truth and automated (${{\text{MVnet}}_{\text{Mixed}}}$) or a pair of second observers manual annotations. *MV* mitral valve

Additional movie files demonstrated MV tracking in cines from the Yale test set (Additional file 1) and in the Lund data set (Additional file 2), using the automated annotations by ${{\text{MVnet}}_{\text{Mixed}}}$. Additional files also show further analysis of the inter-pipeline variability (Additional file 3) and clinical-metric agreement of LV s’ and LV a’, compared with the inter-observer variability (Additional file 4).

## Discussion

In this work, we proposed a dual-stage residual learning framework, MVnet, for time-resolved annotation of the MV in two-chamber and four-chamber views from standard long-axis cine CMR images. The proposed method was fast, fully automated and showed excellent agreement with manual annotation by expert readers in term of valve points positioning as well as with the subsequently extracted LV function parameters. Tedious manual labor is not needed, reducing the processing time from 8 to 20 minutes to 5 or 1 second with batch processing. This enables fully-automated, accurate, fast, and reproducible assessment of LV function in clinical routine. Moreover, this method can be applied retrospectively to any two and four-chamber image acquisition, which are routinely acquired in a standard CMR exam. Additionally, we have systematically shown the advantages and pitfalls of using a single-center, single-vendor, single-expert dataset compared with a multi-center, multi-vendor, multi-expert dataset.

The technical contribution of this work includes the accuracy improvement provided by the second stage. While both networks were trained under the same domain and task, meaning each of them predicts two pairs of coordinates in a given image, the second network processes only highly standardized images, obtained by applying the proposed linear transformation. We showed how much each additional stage, up to one iteration (stage 1+2+2), improved the overall performance. While this assessment could be performed with more iterations (e.g., 1+2+2+2), the accuracy does not further improve, as evaluated in our proof-of-principle work [19]. This adoption of a dual-stage deep learning pipeline in biomedical applications has recently gained some interest to compensate for the technical limitation of one single model, even when trained with a large amount of data. For instance, some dual-stage pipelines with a segmentation task [39, 40] first localize the region of interest with a bounding box and then segment the bounded image, which improved the accuracy from single models. One limitation for an approach consisting of two different tasks is that an error for the localization task can hamper the performance. In our case, we employed the same annotation task for both stages, with a good accuracy on the first stage and an increased performance with the second one. The first stage is enriched by the large-scale study, from different centers, vendors and observers, and data augmentation to further increase the sample diversity [41] allowing model generalization [42]. This approach is commonly employed by one-stage deep learning applications where it is believed that data diversity will solve the image processing task. The specific technical contribution of our work is that we used the benefit of data diversity but also further increased the accuracy by using these results to standardize the images in a novel way.

Our proposed method achieved human-level performance with high robustness and consistency across centers, vendors and observers in a diverse range of conditions. The value of including images from different centers, vendors and conditions aids the generalization of the trained model through seeing all potential variations of the images in a real case scenario [42], whereas including different observers reduces the bias of a single observer [43]. Although this is not the first data diversity study in CMR, as another multi-vendor, multi-center study [44] also evaluated the incremental training strategy for a LV segmentation task with a thorough assessment, our work additionally assessed the annotation pattern diversity. We showed how a model trained on one center could yield a high accuracy in its own dataset but underperformed in datasets from different centers, to achieve generalizable multi-center development [37], and how a different annotation pattern generated discordance against another pattern, even with diverse training inputs.

We evaluated the performance against ground truth with a wide range of parameters including the Euclidean distance, angular distance, MV displacement, MAPSE and LV e’. We showed that the proposed pipeline yielded high reproducibility in a very demanding task with excellent ICC for MV displacement, MAPSE and LV e’, and how the Euclidean distance error may be misleading. This error discrepancy between training labels and clinical metrics has been noted by others. A recent learning-based approach for myocardial segmentation on T1 maps [45] achieved a near-perfect accuracy on estimating T1 values of LV myocardium, even while the segmentation accuracy only yielded a Dice similarity coefficient [46] of 0.85. Although this metric could be misleading, the clinical-metric agreement prevails over image processing performance, as the most important metric. In our study, we showed how a Euclidean distance error could be higher, but the MV plane displacement error was lower.

### Limitations

One limitation of our study is the lack of consensus on how to annotate the MV points as a specific pattern would have homogenized the bias and yielded a lower error. However, our thorough analysis demonstrated that the difference in the annotation patterns did not hamper the performance but instead the MVnet reduced such biases and learned a consensual pattern from the diverse observers, confirming the value of multiple observers for a deep learning application [43]. Another limitation of our study is the missing benefit from incorporating a recurrent neural network architecture to learn spatiotemporal dependencies across the cardiac cycle instead learning from one temporal frame at a time. However, the technical contribution of this work relies on the automated image standardization algorithm to boost both image processing performance and clinical metric agreement, implementing this pipeline to a recurrent neural network architecture may also benefit its learning.

The clinical value of this work is that it provides an automated method for MV plane motion, including established metrics such as MAPSE and LV e’, and also utility for slice-following applications, automated cardiac rest-period identification, among others. Additionally, it does not need any added work in the clinical routine and the post-processing cost is negligible.

## Conclusion

MVnet is a deep learning approach for automated delineation of MV points for MV plane displacement evaluation in CMR long-axis cine images. The method is able to track the MV points, accurately, rapidly and consistently. This will improve the feasibility of CMR methods which rely on valve tracking, such as measurement of e’, or slice-following phase-contrast, and increase their utility in a clinical setting.

## Supplementary Information

**Additional file 1.** Mitral valve tracking in a Yale test sample by MVnet.

**Additional file 2.** Mitral valve tracking in a Lund test sample by MVnet.

**Additional file 3.** Accuracy heatmap of inter-network variability. Each model (MVnet) trained on each training set was compared against other models on the mixed test set by the mean **a** Euclidean and **b** angular distance error, and the agreement with ICC in **c** MV displacement, **d** MAPSE, and **e** LV e'. *ICC* intra-class correlation coefficient, *MV* mitral valve, *MAPSE* mitral annular plane systolic excursion, *LV* left ventricle.

**Additional file 4.** Clinical-metric agreement of LV s' (first row) and LV a' (second row) between an expert manual annotation (or observer 1) and **a** the automated method, and **b** annotation by a second group of observers, on a test set of 50 subjects. One observer from the Yale dataset and another from the Lund dataset annotated 25 subjects from each test set. In each scatter plot the black line denotes the identity line, whereas in each Bland-Altman plot, the red line denotes the mean difference (bias) and the two light dotted lines denote ± 1.96 standard deviations from the mean. *LV* left ventricle.

## Data Availability

The implementation of MVnet will be made freely available in the software Segment (http://www.medviso.com/segment). Imaging data can not be shared due to data privacy consideration details. Other data in the paper will be made available upon reasonable request.

## Abbreviations

2Ch: 2-Chamber
4Ch: 4-Chamber
CMR: Cardiovascular magnetic resonance
e': Early diastolic velocity
ICC: Intra-class correlation coefficient
LV: Left ventricle/left ventricular
MAPSE: Mitral annular plane systolic excursion
MV: Mitral valve
