# Supplementary material for: MVnet: automated time-resolved tracking of the mitral valve plane in CMR long-axis cine images with residual neural networks: a multi-center, multi-vendor study
Source: J Cardiovasc Magn Reson. 2021 Dec 2;23:137. doi: 10.1186/s12968-021-00824-2 (PMC8638514; doi:10.1186/s12968-021-00824-2)
Supplement: Supplementary file 3 — Additional file 3. Accuracy heatmap of inter-network variability. Each model (MVnet) trained on each training set was compared against other models on the mixed test set by the mean a Euclidean and b angular distance error, and the agreement with ICC in c MV displacement, d MAPSE, and e LV e'. ICC intra-class correlation coefficient, MV mitral valve, MAPSE mitral annular plane systolic excursion, LV left ventricle. [file 12968_2021_824_MOESM3_ESM.pdf]

(a) Euclidean distance (mm)

MVnet	Yale	Lund	Mixed
Yale		4.93	3.83
Lund	4.93		1.77
Mixed	3.83	1.77	

(b) Angular distance (º)

MVnet	Yale	Lund	Mixed
Yale		5.32	4.34
Lund	5.32		1.67
Mixed	4.34	1.67	

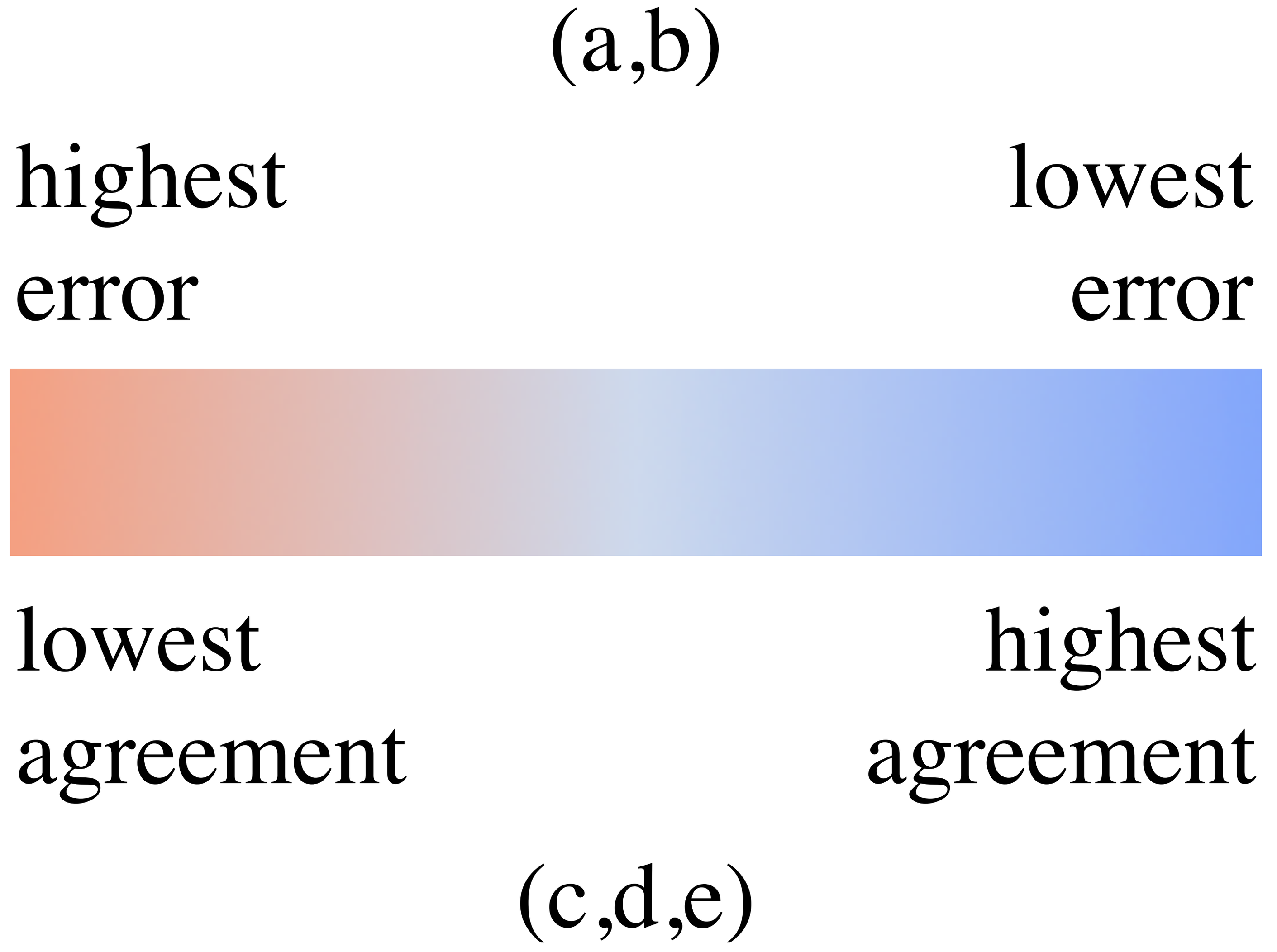

(c) MV displacement (ICC)

MVnet	Yale	Lund	Mixed
Yale		0.93	0.95
Lund	0.93		0.99
Mixed	0.95	0.99	

(d) MAPSE (ICC)

MVnet	Yale	Lund	Mixed
Yale		0.79	0.84
Lund	0.79		0.97
Mixed	0.84	0.97	

(e) LV e’ (ICC)

MVnet	Yale	Lund	Mixed
Yale		0.80	0.85
Lund	0.80		0.93
Mixed	0.85	0.93	
