# Supplementary figures and images for: MVnet: automated time-resolved tracking of the mitral valve plane in CMR long-axis cine images with residual neural networks: a multi-center, multi-vendor study
Source: J Cardiovasc Magn Reson. 2021 Dec 2;23:137. doi: 10.1186/s12968-021-00824-2 (PMC8638514; doi:10.1186/s12968-021-00824-2)

(a) Automated vs Manual measurement

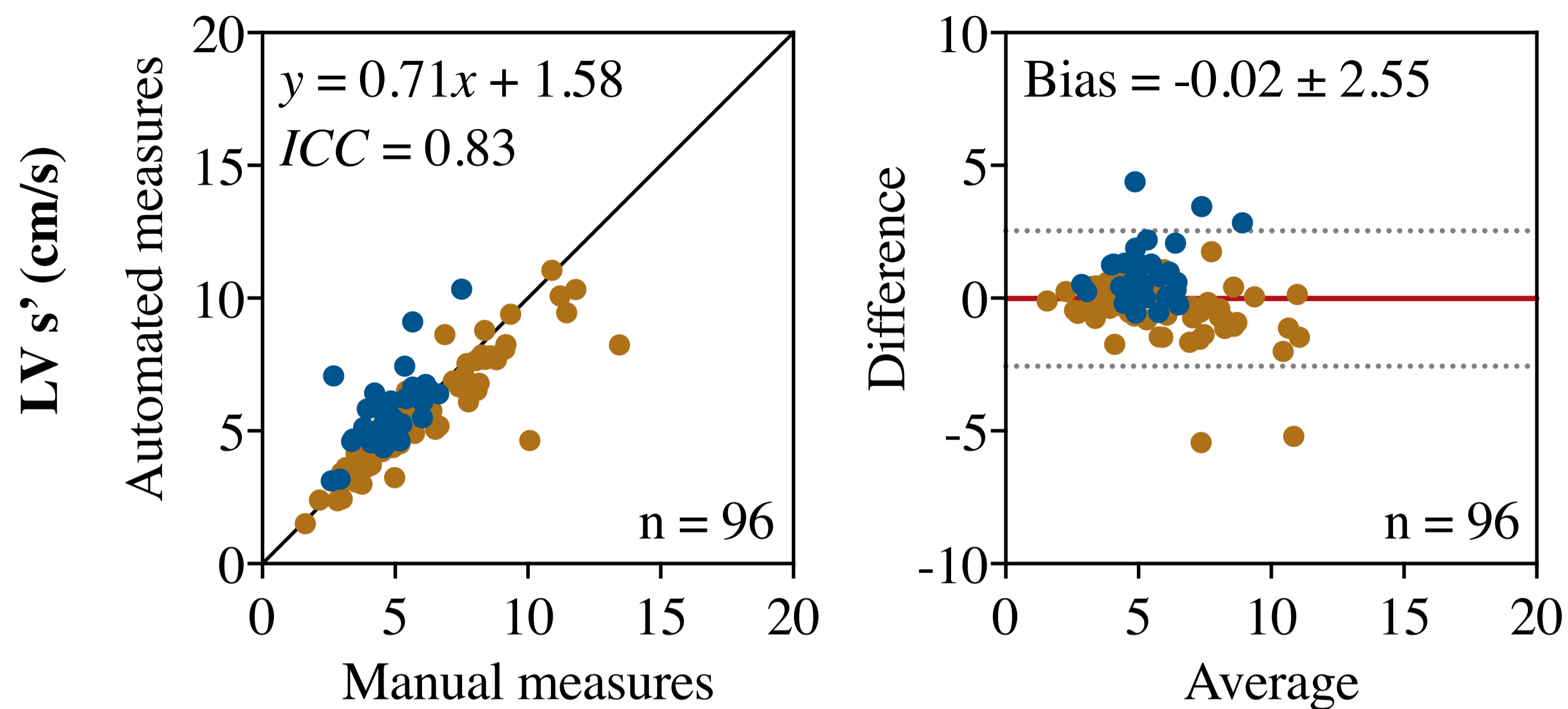

(b) Observer 2 vs Observer 1 measurement

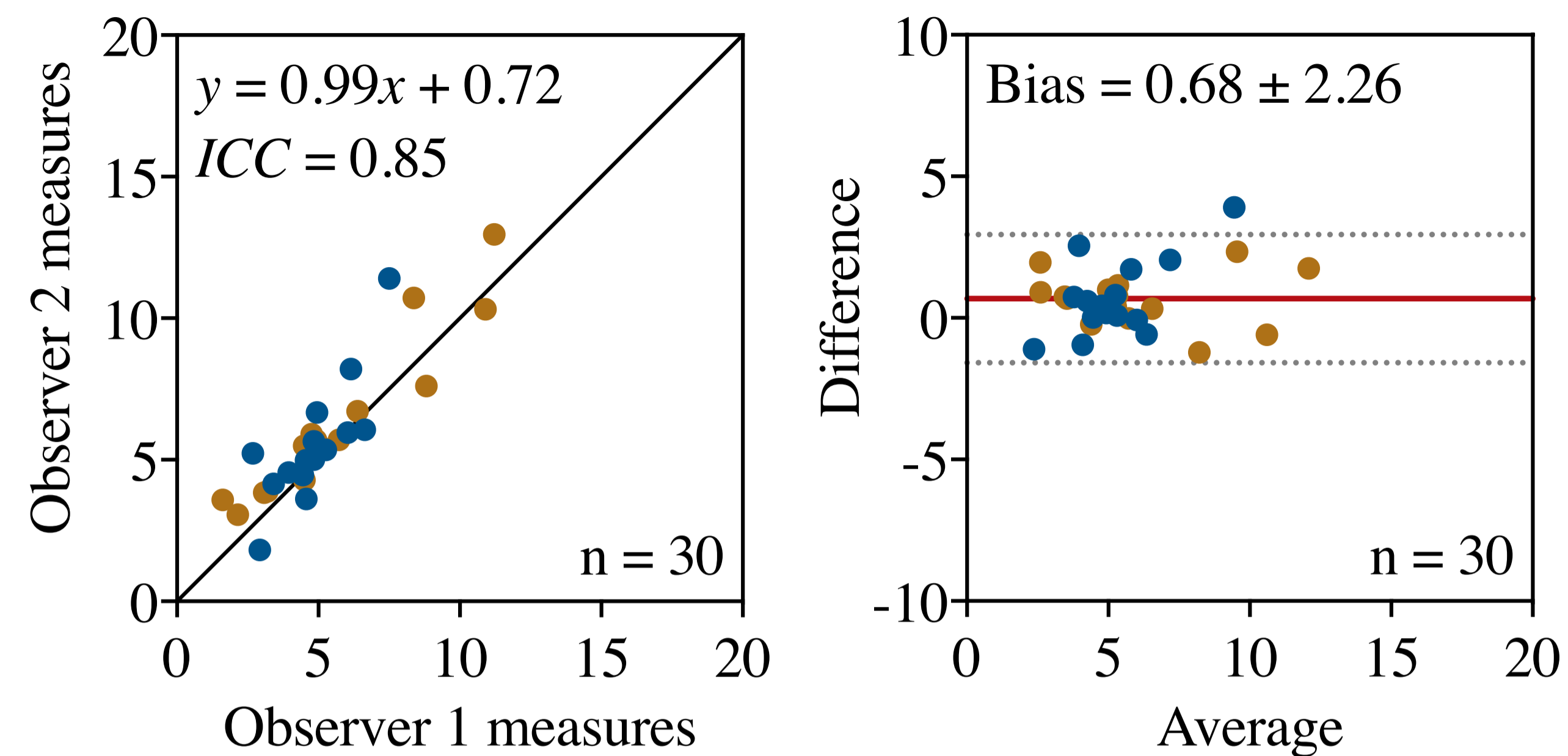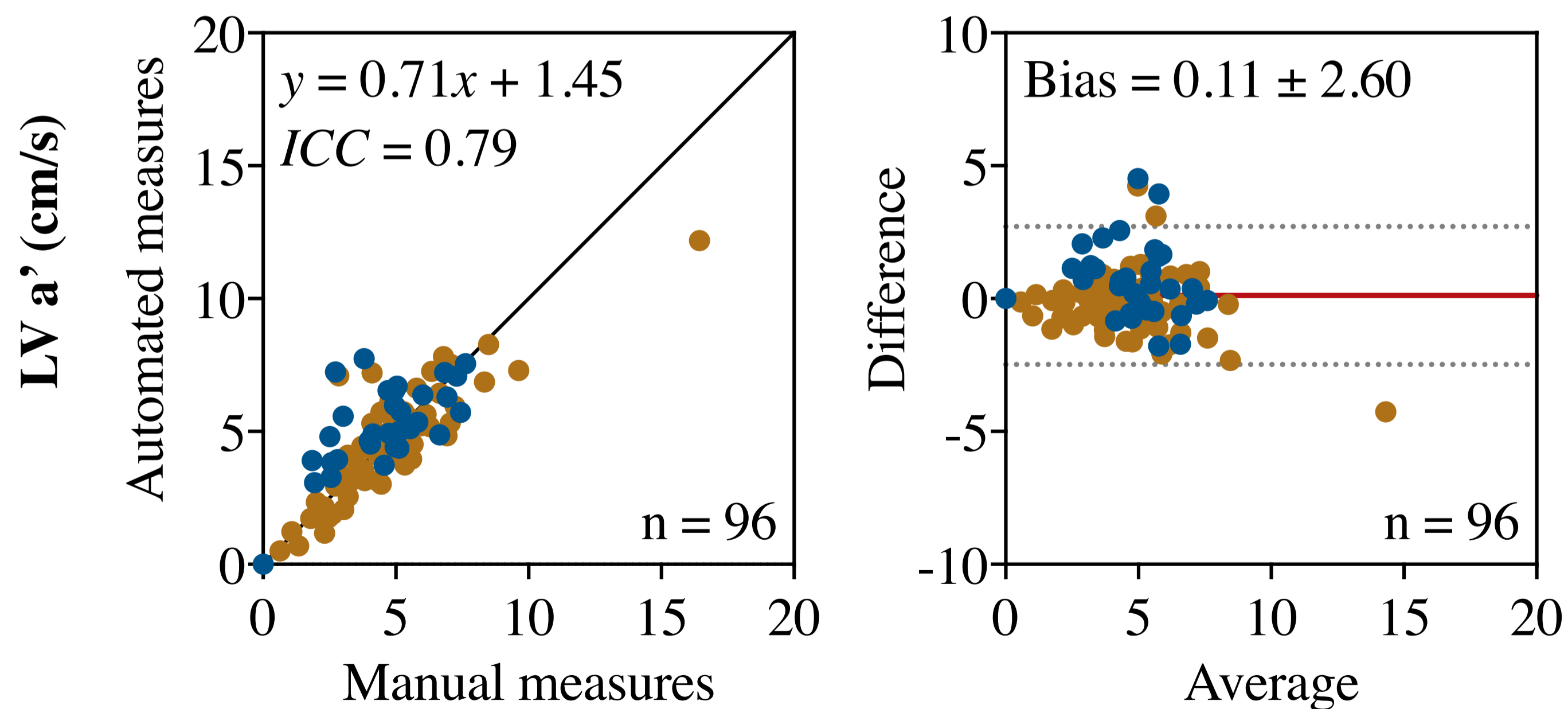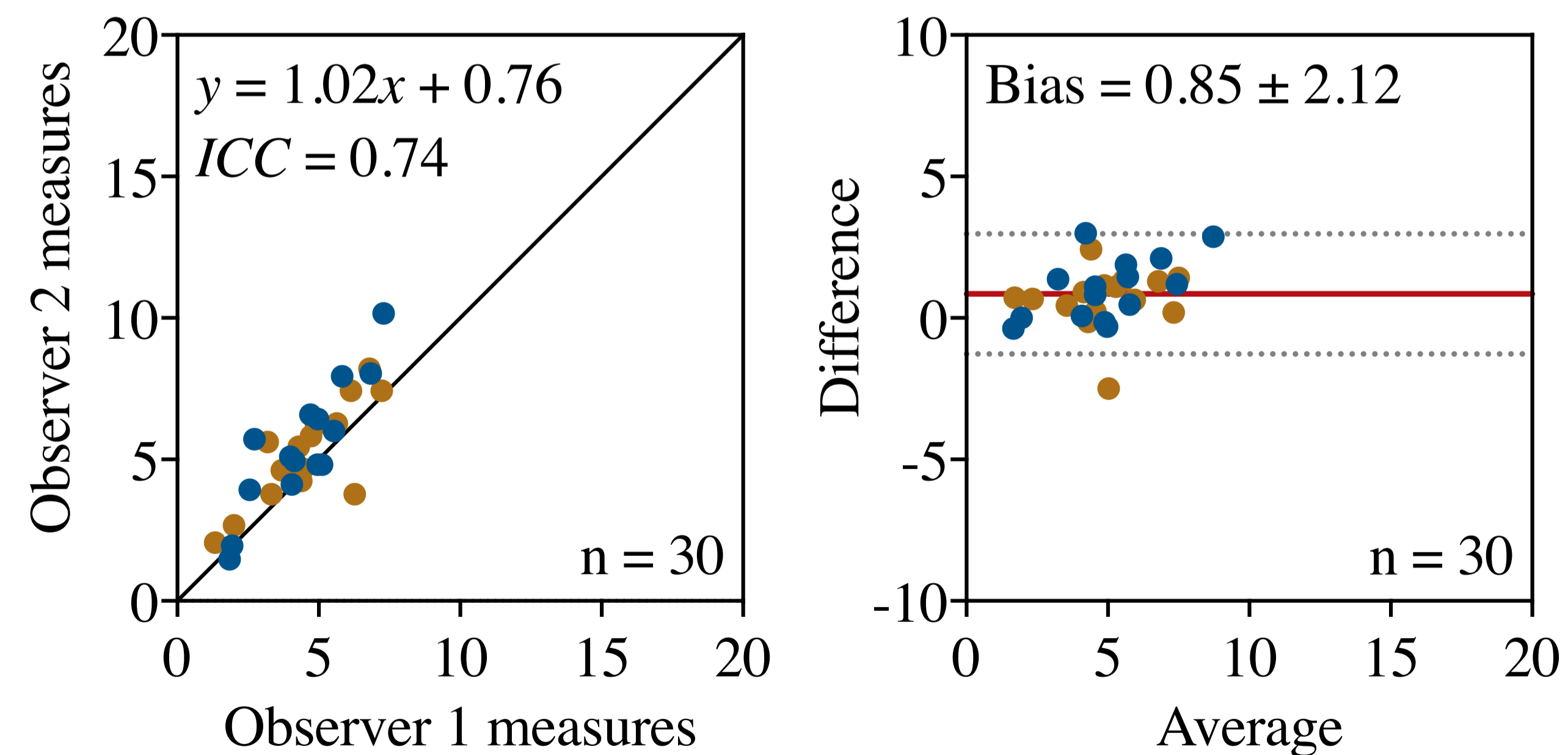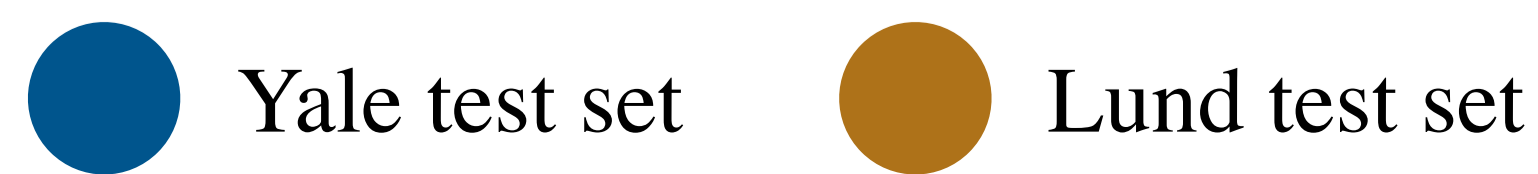

Supplement: Supplementary file 4 — Additional file 4. Clinical-metric agreement of LV s' (first row) and LV a' (second row) between an expert manual annotation (or observer 1) and a the automated method, and b annotation by a second group of observers, on a test set of 50 subjects. One observer from the Yale dataset and another from the Lund dataset annotated 25 subjects from each test set. In each scatter plot the black line denotes the identity line, whereas in each Bland-Altman plot, the red line denotes the mean difference (bias) and the two light dotted lines denote ± 1.96 standard deviations from the mean. LV left ventricle. [file 12968_2021_824_MOESM4_ESM.pdf]
